# Evidence of emergent practice: Teacher candidates facilitating historical discussions in their field placements

**DOI:** 10.1016/j.tate.2018.12.014

**Published:** 2019-04

**Authors:** Abby Reisman, Peter Cipparone, Lightning Jay, Chauncey Monte-Sano, Sarah Schneider Kavanagh, Sarah McGrew, Brad Fogo

**Affiliations:** a University of Pennsylvania, United States; b University of Michigan, United States; c Stanford University, United States; d San Francisco State University, United States

## Abstract

• Teacher candidates can facilitate text-based discussion when prepared.
• Instructional scaffolds can assist candidates in facilitating discussion.
• Assignments, questioning sequences, and prepared materials can support enactment.
• Candidates still struggle to connect discussion to lesson's learning goal.

A growing number of practitioners and researchers of teacher education are calling for the professional preparation of teachers to move closer to practice as a way of connecting teacher education coursework more closely to fieldwork and preparing teachers to enact core instructional practices that engage students in higher-level thinking (Ball & Cohen, 1999; Ball & Forzani, 2009; Grossman, Hammerness, & McDonald, 2009; Lampert et al., 2013). Core practices are content-specific instructional activities that consist of strategies, routines*,* and moves that can be unpacked and learned by teachers (Grossman, Compton, et al., 2009; Grossman, Hammerness, et al., 2009). The focus on practice arises out of a persistent problem in the field: even when teachers have access to professional learning experiences, a gap remains between the kind of instruction that they can envision and the kind of instruction that they can enact (Kennedy, 1999). Practice-based teacher education (PBTE) attempts to address this problem by developing teachers' *practice* as a vehicle for building their vision of powerful instruction (Kavanagh & Rainey, 2017; Windschitl, Thompson, & Braaten, 2011).

As members of the Core Practice Consortium, a group of teacher educators and teacher education researchers spanning 11 institutions (http://corepracticeconsortium.com/), we have been studying how to design methods instruction that supports candidates’ learning and enactment of core teaching practices. We base our work on the assumption that instructional tools—both practical and conceptual—mediate what and how novices learn about teaching (Grossman, Smagorinsky, & Valencia, 1999). A key feature of our work, then, has been to define and specify the components of core instructional practices and design instructional tools that support novices in identifying, adapting, and enacting them.

In this paper, we study teacher candidates’ enactment of one high-leverage practice, text-based discussion, a practice that has been established as valuable to students and challenging for novice teachers. In particular, we examine how teacher candidates (N = 32) from two different practice-based social studies methods courses—one for elementary teachers and one for secondary—facilitated discussion in their field placements. We ask:

(a) How did teacher candidates enact discussion in their field placements after being introduced to discussion through a practice-based approach in their methods course?
(b) How and to what extent did teacher candidates' discussion facilitation vary depending on the scaffolds (e.g., lesson structure, instructional materials) they received in methods instruction?

Our goals are three-fold. First, we hope to contribute to the growing body of empirical studies that shed light on what teacher candidates take away from practice-based instruction as they begin to teach (Kloser, Wilsey, Madkins, & Windschitl, in press; Reisman et al., 2018; Windschitl, Thompson, & Braaten, 2011). Second, by comparing elementary and secondary discussions using the same analytic framework, we hoped to determine whether our specification for discussion constituted a stable and observable instructional practice across age groups. Third, by identifying, naming, and cataloguing the types of moves that candidates use when attempting to facilitate discussion, we hope to contribute to a “common technical vocabulary” of instruction (Grossman & McDonald, 2008, p. 186), so that practitioners and researchers might have common points of reference as they work to construct shared visions of instruction across educational contexts.

## 1 Discussion in history classrooms

A substantial body of research links classroom discussion to student outcomes ranging from student engagement to conceptual understanding to subject matter learning (e.g., Engle & Conant, 2002; Kazemi & Stipek, 2001; Nystrand, 1997). In social studies, researchers have theorized that classroom discussion can advance the democratic purposes of schooling (Hess, 2009; Levinson, 2012; Parker & Hess, 2001), and recent research has found effects for discussion on student political knowledge and involvement (Hess & McAvoy, 2015). Despite these benefits, discussion remains stubbornly absent across the curriculum, especially in history classrooms that emphasize rote memorization and textbook work (e.g., Reisman, 2015; Bain, 2006; Saye & SSIRC, 2013).

Discussions may be persistently absent from classrooms because they are so pedagogically complex. To support and orchestrate student discourse in service of subject matter learning, a teacher must craft questions that open the content to investigation, and employ pedagogical moves that initiate students into sophisticated epistemological work (Ball & Forzani, 2009; Grossman, Hammerness, et al., 2009; Shulman, 1986). Social studies researchers have identified entry points for preparing novices to facilitate discussion. Parker (2003, 2006) underscored the importance of helping novices select and prepare texts, establish norms, and foster—in themselves and their students—stances of humility and reciprocity that undergird good listening. Hess (2004) used classroom video to help teacher candidates broaden their vision of students' capacity to engage in discussions. All these interventions focused on developing teacher candidates’ discursive repertoires beyond the Initiation-Response-Evaluation pattern that typifies classroom instruction (Cazden, 2001).

While the literature on practice-based instruction aims to provide language that can apply across content areas, thus far it focuses on discussion facilitation in other subjects (see Ghousseini, 2009; Kavanagh & Rainey, 2017; Pimentel & McNeill, 2013). Comparable work on teaching historical discussions remains in its infancy. Below we sketch our efforts to design practice-based instruction in two teacher education contexts, then present our analysis of whether and how participating teacher candidates showed evidence of learning from this instruction in their field placements.

## 2 Practice-based instruction on facilitating discussions in history

For this study, we focused on the elementary social studies methods course at University A and the secondary social studies methods course at University B over two academic years, 2014–2015 and 2015–2016. We collaboratively designed and studied our instruction around document-based historical discussion, a process informed by our participation in the Core Practice Consortium.

In keeping with the commitment of practice-based teacher education, we began with a shared specification of the practice of document-based historical discussion that drew on existing scholarship (e.g., Nystrand, Wu, Gamoran, Zeiser, & Long, 2003; Parker, 2010). We defined *text-based, whole-class discussion in history classrooms* as those activities in which the teacher and students negotiate historical questions using each other's ideas and historical texts as resources. The purposes of such discussions are to build collective knowledge and allow students to practice engaging in historical interpretation. In productive discussions, the teacher and a wide range of students contribute orally, listen actively, and respond to and learn from others' contributions. We also shared an insistence on keeping disciplinary reading at the center of discussion (Shanahan, Shanahan, & Misischia, 2011). Using this specification of discussion, we developed our methods instruction around core pedagogies of teacher education proposed by Grossman, Compton, et al. (2009) and Grossman, Hammerness, et al. (2009): we presented candidates with multiple *representations* of the practice to observe and study; we provided a shared language to aid them in *decomposing* the practice into its constituent parts; and we created opportunities for them to *approximate* the practice in contexts reflecting varying degrees of authenticity.

Despite these shared conceptual underpinnings, our courses differed due to their programmatic contexts. Given the cross-program emphasis on high-leverage practices at University A, candidates in the elementary social studies methods course had worked in other courses on posing questions, explaining, and eliciting, probing, and developing students' ideas about content. The teacher educator framed discussion both as a distinct core practice and as one that incorporated these other practices. To scaffold facilitation of whole-class, text-based historical discussion, the teacher educator embedded the practice in a “visual inquiry lesson” (Johnson, 2013), in which students examine and compare two related images (e.g., a historical and contemporary image of an automobile factory or contrasting depictions of the Boston Tea Party) in order to answer an interpretive central question. Discussion was divided into three sections: (a) the teacher prompted students to share what they noticed about the images; (b) students made inferences about what they thought was happening in the images; (c) students answered the central question by comparing the images. The teacher educator relied on Beck and McKeown's (2006) terminology to decompose discussion, including *marking* (calling attention to certain ideas), *turning back to students and to text* (directing students to text or to each other), *annotating* (adding information), *revoicing* (restating student ideas), *recapping* (summarizing), and *modeling* (demonstrating expert thinking with text). During candidate rehearsals of discussion, teacher educator feedback focused on helping candidates frame open-ended questions, use text-based and student-to-student talk moves, and other considerations such as using age-appropriate language and providing wait time.

In the secondary methods course at University B, discussion was presented as the activity structure at the end of a document-based lesson, rather than the structure of the entire lesson. Discussion occurred after students had read and answered questions about individual documents (Reisman, 2012), when the teacher brought students into the “historical problem space” (Reisman, 2015) to grapple with the strangeness of the past and the challenges faced in attempting to understand it. In this methods course, representations of discussion were decomposed on two levels. First, teacher candidates examined whether and how the facilitator connected discussion of each of the documents to the central historical question. Second, teacher candidates analyzed specific facilitation moves using the following terms: *textual press* (asking students to substantiate claims), *uptake* (following up on a student's textual reference with a question), *modeling* (demonstrating how to use text to support a historical claim), *marking text* (directing students' attention to a document and asking an interpretive question about it), *revoicing* (reformulating a student's claim to highlight the relationship between the claim and warrant), and *stabilizing historical content* (reviewing critical background knowledge) (Beck & McKeown, 2006; Nystrand, Gamoran, Kachur, & Prendergast, 1997; O'Connor & Michaels, 1993; Reisman, 2015). During rehearsals, in addition to offering feedback on these moves, the teacher educator helped candidates identify which aspects of the document were most generative in responding to the central historical question.

**Fig. 1:**
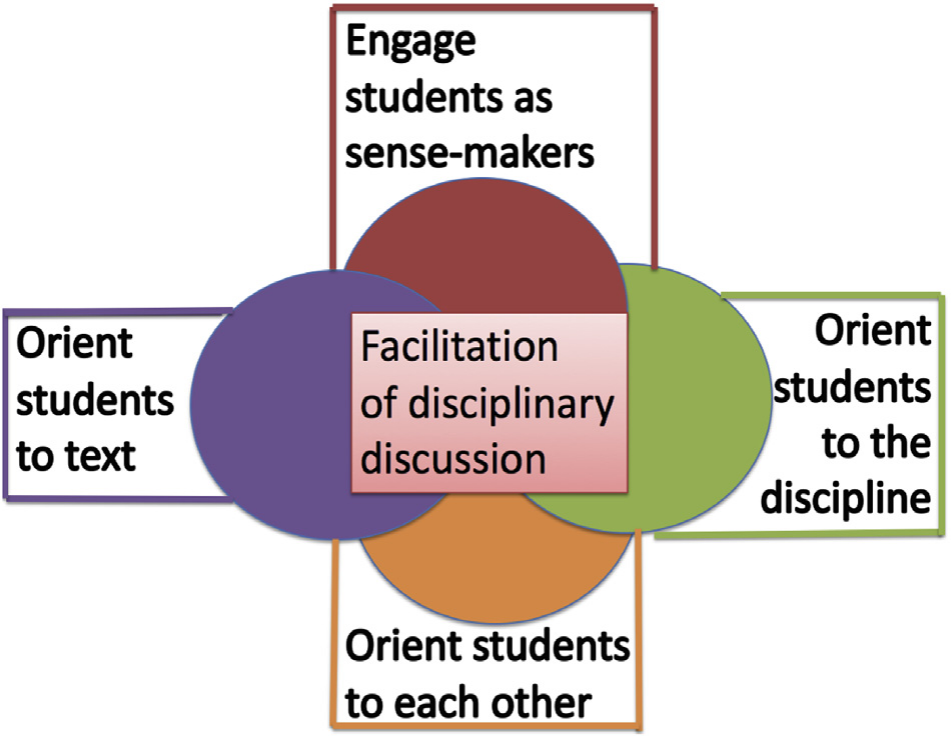
A framework for facilitating historical discussions.

In the second year of the study, benefitting from ongoing discussion and data analysis with members of the Core Practice Consortium, the teacher educator in the secondary methods course incorporated the Framework for Facilitating Historical Discussion (see next section and Fig. 1 below) as a conceptual tool to frame and debrief discussion facilitation.

## 3 Framework for Facilitating Historical Discussions

In previous work (Reisman et al., 2018) we discussed an analytic framework that resulted from our initial review of candidates’ discussion facilitation videos. Intended as a conceptual rather than empirical contribution, the Framework for Facilitating Historical Discussions (see Fig. 1) represents what we believe to be the stable components of the practice that transcended lesson structures (e.g., visual inquiry lesson or document-based lesson) and classroom contexts (e.g., elementary versus secondary). The framework comprises four prongs, each of which represents a sub-practice: (a) engaging students as sense-makers; (b) orienting students to each other; (c) orienting students to the text; and (d) orienting students to the discipline. This framework was used as an instructional tool in the secondary course for year two of this study, but not in the elementary course. In this paper, we leverage this framework to help structure our analysis.

Our initial review also identified ways candidates across contexts succeeded and struggled to enact each sub-practice. For example, in both elementary and secondary videos, candidates succeeded in orienting students to the text but struggled to move beyond low-level questions (e.g., “What do you notice?” or prompts to summarize text), and to support students’ interpretation of texts in light of a larger historical question. We also found candidates rarely oriented students to each other. Finally, we had difficulty identifying examples of candidates orienting students to the discipline, despite a shared insistence in both courses that discussion advance disciplinary learning and promote disciplinary reading practices that allow historians to render plausible interpretations of the past.

The process of creating the Framework for Facilitating Historical Discussions and observing the first year of data served as an analytic starting place to systematically examine the full set of candidate videos in this study. Our intent is to identify how candidates facilitated discussion and where they appeared to struggle. Additionally, we examined whether and how instructional scaffolds supported candidate facilitation.

## 4 Method

### 4.1 Study context

We analyzed footage of two cohorts of teacher candidates (2014–2015 and 2015–2016) from two teacher education programs, an elementary social studies methods course at University A and a secondary social studies methods course at University B, as they facilitated discussion in their field placements (N = 32). Teacher candidates in the elementary program at University A came from a variety of undergraduate majors and enrolled in the social studies methods course in the second semester of a 12-month master's program. The course was taken concurrently with other subject area methods and foundations courses, all of which shared a focus on core teaching practices. Students in the secondary methods course enrolled in a two-semester sequence as part of a 12-month master's program. Instruction on classroom discussion occurred during the second semester. Candidates entered the program with an undergraduate major in history or at least four courses in history and five additional courses in the social sciences, a state requirement for social studies certification.

The social studies methods courses were taught by the first and fourth authors of this paper. In occupying both roles we blurred the line between research and practice in ways that inevitably shaped the study (Peshkin, 1988). Nonetheless, we believe our dual perspectives as teacher educators and authors allowed us to draw a detailed portrait of how teacher candidates enacted the core practice of discussion. We also took a number of steps to ensure validity as we analyzed the data. For example, authors who were not lead teacher educators coded all data, a process described below.

**Elementary discussion assignment*.*** Candidates were instructed to plan and facilitate a lesson in their field placements and to post the entire video (15–40 min, depending on grade level) to an online video platform, along with their lesson plan and a one-page reflection on their teaching, students’ thinking, and next steps. We used the platform *Edthena*, which allowed candidates to share videos with instructors and cohort members who could comment and annotate. In Fall 2014, 7 of 11 study participants used instructor-created curricular materials; the remaining 4 students used materials they designed themselves. In the second year, only 1 of 5 study participants used instructor-created curricular materials.

**Secondary discussion assignment*.*** Teacher candidates were instructed to post a 5–10 min video of themselves “facilitating a discussion around historical texts” on *Edthena*, along with any contextual information or materials that would help their classmates understand the video. Each candidate posted comments on classmates’ videos and was encouraged to refer to facilitation moves discussed in class. Candidates also submitted a one-page reflection about the discussion and what they might have done differently. Half of the 16 secondary candidates (4 from Year 1; 4 from Year 2) used instructor-created curricular materials. The other half designed materials themselves.

### 4.2 Data sources

Our analysis focuses on the 32 videotaped discussions candidates submitted to *Edthena* as part of the course assignment at each site (see Table 1). Because our primary interest is in the transfer from instruction to the classroom, we chose to rely solely on video evidence for this study. We analyzed videos of discussions led by 16 elementary teacher education students (11 from Year 1, 5 from Year 2) and 16 secondary history teacher education students (7 from Year 1, 9 from Year 2). The elementary sample included all teacher candidates in both years who taught fourth- and fifth-grade students and whose discussions centered on historical content or content involving contemporary issues rooted in history (e.g., Native American sports mascots). Teacher candidates in kindergarten through third grade classrooms were removed from the data set because their lessons centered on personal or relational questions such as “Which photo shows a situation where you should ask for help from an adult?” The secondary sample included all teacher candidates in seventh-grade through 12th-grade classrooms whose field placements were in history classrooms. Like in the elementary sample, we included videos focused on historical content as well as those that focused on contemporary issues rooted in history (e.g., Should the Elgin Marbles be removed from the British Museum and returned to Greece?). In the secondary setting, teacher candidates cropped their videos to include just 5–10 min of whole-class discussion. We did not have access to the remainder of their videos. In the elementary setting, teacher candidates submitted their entire class session. Reasoning that historical thinking is fundamentally intertextual (see Collingwood, 1946; Shanahan, Shanahan, & Misischia, 2011; Wineburg, 1991), we began our analysis of elementary videos when the teacher candidate introduced the second historical image and prompted students to make connections between two images. If the candidate prompted students to answer the central historical question, we coded that segment, too.

**Table 1:** List of candidates’ submitted videos for edthena assignment.

Elementary Videos				
Candidate ID	Field Placement Grade Level	Lesson Topic	Instructor-created	Video Length (Min)
E1-1^a^	4	Assembly line	Yes	18
E1-2	5	Native American tribes	No	14
E1-3	5	Native American/European interactions	No	12
E1-4	5	Christopher Columbus/Native American relations	No	20
E1-5	4	Michigan Maps	Yes	22
E1-6	5	Stamp Act	Yes	12
E1-7	4	Assembly line	Yes	13
E1-8	5	Christopher Columbus/Native American relations	No	18
E1-9	4	Michigan Maps	Yes	21
E1-10	5	Boston Tea Party	Yes	16
E1-11	4	Michigan Maps	Yes	22
E2-1	4	Michigan Maps	Yes	7
E2-2	5	Native American mascots	No	18
E2-3	5	Carlisle Indian School	No	15
E2-4	5	Christopher Columbus/Native American relations	No	11
E2-5	5	Native American mascots	No	16
Secondary Videos				
Candidate ID	Field Placement Grade Level	Lesson Topic	Instructor-created	Video Length (Min)
S1-1	9	John Locke	No	7
S1-2	10	Cold War	No	7.5
S1-3	11	Same-sex marriage	No	7
S1-4	9	Nat Turner	Yes	5
S1-5	11	Montgomery bus boycott	Yes	3.5
S1-6	11	Social Security/New Deal	Yes	8
S1-7	10	Abraham Lincoln	Yes	6.5
S2-1	8	Inca origin story	No	17.5
S2-2	11	Syrian refugees	No	7
S2-3	11	Homestead Strike	Yes	6.5
S2-4	8	1950s Redevelopment of West Philadelphia	No	6
S2-5	10	Elgin Marbles	No	9
S2-6	8	Pocahontas	Yes	5
S2-7	10	Indian Removal Act	Yes	8
S2-8	8	First Amendment protections	No	10
S2-9	7	Abraham Lincoln	Yes	9

a Candidates are identified by program and cohort, and then by an arbitrary number. For example, E1-11 is an elementary candidate from YR1 who was assigned ID number 11.

### 4.3 Data analysis

We used Studiocode video-analysis software to analyze teacher candidate videos. We coded teacher candidate facilitation at the level of the utterance, which we regarded as equivalent to a turn, beginning with the start of speech and ending when another person spoke. All instances were coded with as many labels as applied. We did not code student talk in this analysis beyond calculating the number of student turns per minute.

We used the Framework for Facilitating Historical Discussions (Fig. 1) to organize and characterize our codes. We began with a list of terms we used in our methods instruction to describe discussion facilitation moves, as well as relevant terms drawn from the literature on discussion (e.g., Kazemi & Cunard, 2016). These included disciplinary terms such as “sourcing” and “contextualization” and terms that pertained to other prongs of the framework. For example, “textual press” was included as a subcode under “orienting students to the text,” while “asking students to respond to students” served as a subcode under “orienting students to each other.” After developing an initial set of codes, we culled them to eliminate redundancies. For example, we eliminated *uptake*, defined in the literature as following up on a student's comment with a question (Nystrand, 1997), because we determined that *uptake* could appear under multiple prongs of the framework (e.g., by prompting students to substantiate their claim with evidence [orienting to text] or by asking another student to respond [orienting to each other]). Table 2 lists our final codes. We applied codes in the fourth prong— “Engaging Students as Sense-makers”—only when candidates initiated elicitations, for example by asking students what they noticed in a particular image or whether they found a particular document reliable, and each initial elicitation was characterized as either “open” or “closed” (see Kavanagh & Rainey, 2017; pp. 916-17, for similar coding scheme). All subsequent elicitations in which a candidate followed up on that topic were coded using the remaining three prongs of the framework. All videos were double coded by two researchers and disagreements were resolved through discussion.

**Table 2:** Discussion facilitation codes.

Code	Description	Labels	Examples
Engage Students as Sense-makers	Teacher poses question(s) that elicit student voice and initiate new conversation thread.	Open Closed **Sub-labels:** Prompt noticing Prompt inference Prompt interpretation of single document Pose central historical question	“Let's move onto Document B. Why does Boudinot support Indian Removal?” (Open; **Sub-label:** prompt interpretation of single document)
Orient students to each other	Teacher prompts students to revoice, summarize, elaborate upon, or respond to something another student said; teacher draws a connection between two or more student comments.	Ask SS to revoice S Ask SS to respond to S Connect SS ideas Expose discussion structure Meta-comment about orienting to each other	“Manuel just said____What do you think about that argument?” (Ask SS to respond to S) That's a really different idea than what Simone just put forth…(Connect SS ideas)
Orient students to the text	Teacher prompts students to refer directly to, summarize, or interpret a shared text.	Summarizing Textual Press Marking text Meta-comment about orienting to text	“So you're claiming ______. What's your evidence for that?” (Textual press) “What about the second paragraph? Who is Churchill blaming there?” (Marking text)
Orient students to the discipline	Teacher prompts students to engage in the interpretive work of historical analysis by considering authorship, relevant background knowledge, and available textual evidence.	Source Contextualize Corroborate Highlight Claim-Evidence Stabilize content knowledge Modeling historical thinking Meta-comment on historical thinking	“Who wrote this document? How might that change the way we view it?” (Source) “What was going on at the time that Document B was written?” (Contextualize) “Does Document A give the same account as Document B?” (Corroborate) “So John is arguing that Lincoln is racist because of [textual evidence].” (Highlight claim-evidence) “Okay so let's make sure we've got this clear: how many ships did Columbus come over with? And where did they land?” (Stabilize content knowledge) “I'm wondering what people at the time were thinking when they listened to this speech.” (Modeling historical thinking) “When we look at documents, we always look at the source first…” (Meta-comment on historical thinking)

In our analyses we sought to identify patterns in the candidates’ enactments. We tallied the number of utterances and calculated the percentage labeled with each code to address variation in video length. That allowed us to discern the general proportion of utterances by teacher candidates—across sites and cohorts—that attempted to orient students to the text, each other, and the discipline. When relevant, we also report frequency per minute. We conducted independent-samples *t*-tests to compare programs.

**Inter-program comparisons.** We compared discussion facilitation in the two programs to explore how differences in the *lesson structure* of the assignments influenced candidates' enactment. The elementary candidates were instructed to follow a fairly prescribed question sequence in their discussion facilitation, whereas the secondary candidates were not provided an analogous structure. We wondered whether the elementary candidates enacted the question sequence they were provided and, if so, whether they enacted some parts of the question sequence more than others. We also wondered whether the absence of a question sequence would result in greater variation among secondary candidates’ enactment of discussion.

**Intra-program comparisons.** Within each program, we compared the facilitation of candidates who used instructor-created materials with those who used materials that they created. Instructor-created materials represent an additional way that teacher educators can scaffold and support novice facilitation of discussion. Presumably, such materials would be well-curated, generative, and designed to prompt disciplinary learning. A potential downside of instructor-created materials is that candidates might be less familiar with the learning goal and underlying logic of the lesson. Our analysis sought to pinpoint how the materials influenced candidate facilitation. Across both the elementary and secondary program, candidates who facilitated discussions of contemporary issues created their own materials.

In the secondary program, we also compared candidates' discussion facilitation in Year 1 to Year 2 of the study. In the second year of the study, the teacher educator incorporated the Framework for Facilitating Historical Discussion as a tool to frame and debrief discussion facilitation. The elementary methods instructor did not use this conceptual tool.

## 5 Results

Analysis of candidate videos indicated that teacher candidates regularly enacted many of the facilitation moves that had been the explicit focus of instruction and rehearsal in methods courses. Below we present the frequency with which teacher candidates engaged students in each of the four prongs of the discussion framework. In each section, we report if instructional scaffolds resulted in any observable differences between programs. There was significant variation across candidates and context, but, in general, we saw candidates enact the moves they had practiced within the lesson structures they learned. They oriented students to the text and elicited open discussion at particularly high rates, but were somewhat less likely to orient students to the discipline or to one another, and they rarely used meta-commentary to explain the purpose of the moves to students. We elaborate on these findings below.

### 5.1 Prong 1: Engaging students as sense-makers

Multiple findings indicate that candidates engaged students as sense-makers. First, we found that the bulk of candidates' initial elicitations were coded “open” (92% of elementary candidates' initial elicitations and 81% of secondary candidates’ initial elicitations). This finding indicates that when candidates initiated new threads of discourse, they mostly did so with open-ended questions. Second, we found high student participation across the videos. Whereas teacher utterances occurred at an average frequency of 1.77 per minute in elementary videos—in other words, nearly twice per minute—student utterances occurred at an almost equal frequency of 1.46. In one quarter of the elementary videos, student turns occurred as frequently or more frequently than teacher turns. Likewise, secondary candidate turns (1.87 per minute) were almost matched by the frequency of student turns (1.47 per minute). In nearly half the secondary videos, student turns occurred as frequently or more frequently than teacher turns.

However, a closer look at how and when the central historical question was posed across the videos indicated that both elementary and secondary candidates struggled to engage students in the core interpretive work of text-based historical discussion. According to the structure of the visual inquiry lesson, candidates should have posed the central question in the final segment of the discussion, after students had studied the images and compared them. Six elementary teacher candidates did not pose an interpretive central question, the cornerstone of the visual inquiry lesson plans. We did, however, observe elementary candidates enacting other elements of the visual inquiry lesson. Elementary candidates were more likely than secondary candidates to use open elicitations to prompt students to notice aspects of the images (e.g., What do you see in the image?) (*t*(30) = 8.84, *p* = .000) and to elicit inferences (e.g., What do you think the Native Americans were thinking?) (*t*(30) = 2.83, *p* = .008). But elementary candidates were far less likely to prompt students to *interpret* the visual texts (e.g., How are the Native Americans being portrayed?).

For many of the candidates, pacing was a challenge. Of the 10 (63%) elementary candidates who did pose a central question, four (25%) did so with less than 2 min remaining in the lesson. These findings suggest that the structure of the assignment made it difficult for elementary candidates to engage students in interpretive work. Of the remaining 6 (38%) candidates who posed central questions, only 2 posed historical questions about the images that prompted analysis and interpretation: E1-11 asked students to consider how and why maps of Michigan changed from the 1700s to today, and E2-3 posed a question about the Carlisle Boarding School for Native Americans (“How do these pictures show how entering the school changed or affected their culture?“).

Secondary candidates were more likely to pose the central historical question (*t*(30) = 2.61, *p* = .014) and prompt students to interpret a single document (*t*(30) = 2.50, *p* = .018), reflecting the types of open elicitations one might expect in a document-based lesson discussion. Even here, though, three candidates (19%) never posed a central historical question. Four (25%) secondary candidates posed a central historical question but did not use the question to prompt analysis and interpretation of the documents; instead, they focused discussion on comprehending key points in the documents.

### 5.2 Prong 2: Orienting to the text

An utterance was coded as “orienting to the text” if the candidate directed students' attention to the lesson's texts, either to summarize, interpret a particular passage, or find evidence to support a claim. Over 25% of elementary candidates' utterances oriented students to the text, which in the elementary case meant images. Among these, candidates were most likely to use “textual press” moves, asking students to support their claims by pointing to specific evidence in the images. The second most common facilitation move among elementary candidates was “marking text,” when the candidate asked students questions about specific parts of the image such as, “What do you notice about the numbers [on this map]?” (E1-11). We found that elementary candidates who used instructor-created materials marked the text twice as often as those who created their own materials.

Secondary teacher candidates spent more time orienting students to the text than any other part of the framework. Orienting to the text made up 41% of secondary candidate utterances and occurred almost once per minute. Of these textual moves, the most common was asking students to summarize the text; secondary candidates were significantly more likely to prompt students to summarize than elementary candidates (*t*(30) = 3.02, *p* = .005). This difference underscores the challenges of ensuring comprehension of written text while facilitating discussion. Secondary candidates also commonly prompted students to support their claims with evidence from documents, and just over half of the secondary candidates asked students to parse or interpret *specific* passages of the texts (i.e., marking text). Finally, similar to the elementary candidates, we found that those candidates who used instructor-created materials were more likely to use “textual press” to prompt students to substantiate their claims with evidence (*t*(14) = 2.88, *p* = .012) than their classmates who used materials that they designed.

Despite the high proportion of orienting to text moves in both the elementary and secondary videos, we did not find a single instance of secondary candidates making a meta-comment about the importance of attending to the text in discussion (e.g., “You always want to make sure your classmates know exactly where you're reading from in the document”). Elementary candidates were more likely to make such comments, but these largely described what they saw students doing with the images (e.g., “I really like that Jamie pointed to the details in the picture to support his ideas” (E2-20)), and did not explain *why* such textual engagement was important.

### 5.3 Prong 3: Orienting students to each other

Candidates across both programs were less likely to orient students to each other than to the text or the discipline, though elementary candidates were more likely to do so than secondary candidates. All elementary candidates used at least one “orienting to each other” move, compared to 75% of secondary candidates. In both contexts, candidates primarily relied on two moves: “ask student to respond to student” by prompting students to connect to a prior student's comment, and “expose the discussion structure,” or helping students track and align themselves with the various perspectives raised in the discussion. In the elementary videos, teacher candidates commonly exposed the discussion structure by having students show their agreement or disagreement with thumbs up or down.

Although asking students to respond to each other and exposing the discussion structure were two distinct moves in our coding scheme, we counted 10 instances in which elementary candidates used them together. For example, E1-7, who used this sequence more than once in a lesson comparing two images of assembly lines in auto factories, said, “John said he might be controlling the conveyor belt. Who agrees with that? Thumbs up if you agree … Miguel, why do you agree?” In a lesson that could easily devolve into an incoherent sharing of random observations, this discursive sequence allowed the candidate to focus students' attention and help them build on each other's comments. When used productively, this sequence could also allow a candidate to showcase student comments most relevant to the learning goal.

For the above sequence to be effective, Miguel should explicitly connect his idea to John's. Such follow-up rarely happened in the elementary videos, where students were often more eager to share their own observations than to build on their classmates'. Only two candidates were successful in *returning* to students' comments that they wanted to emphasize. For example, in a lesson comparing historic and contemporary maps of Michigan, a key learning goal was for students to note how each map highlighted the common means of transportation for the time. E1-11 asked students “What do you notice about these red lines?” in the contemporary map. After one student responded with an unrelated observation, a second student suggested that the red lines were roads, at which point E1-11, said, “Who agrees with Dana that the red lines are roads? Put your thumb up if you agree. Who disagrees? Billy why do you have a sideways thumb?” Billy responded that the red lines were only *some* of the roads and then another student made an unrelated observation. At this point, E1-11 *returned* to the initial comment: “I want to go back to Dana's point that the red lines are roads. Kai, what do you want to add?” Later in the discussion, E1-11 pushed students to draw a further inference about how transportation had changed since the 1800s—namely, that the lines in the first map featured waterways rather than roads. Few candidates were as effective in ultimately tying student observations to the larger learning goal. However, many were able to use the discursive sequence of “expose the discussion structure” and “ask student to respond to student” to showcase those comments they deemed most relevant.

Secondary candidates were also less likely to orient students to each other than to the discipline or the text. Although secondary candidates on average relied on the same two moves as the elementary candidates, they exposed the discussion structure in a different way. Whereas elementary candidates prompted students to indicate with their thumbs if they agreed or disagreed with the prior comment, secondary candidates were more likely to revoice the prior student comment in an effort to highlight the competing claims that were on the table. For example, in the following excerpt, students debated the question, “Should the British Museum return the Elgin Marbles to Greece?” and two students spoke on behalf of the British Museum before the teacher candidate intervened:

"Ok, so I just want to clarify that Victor is raising a *new* point, that Greece can still appreciate its culture even if the objects are in the British museum. Okay, I saw a lot of hands. Let's go with Michelle." (S2-5)

S2-6 exposed the discussion structure in a similar way while facilitating a discussion about Pocahontas: “So Mike and Jada and Ricardo are saying that Document A is more reliable because he has no reason to lie because [Powhatan's capture of John Smith] happened the same year he wrote the book. Who disagrees?” In both cases, the candidates first summarized the new argument and then invited other students to respond.

In contrast to the thumbs up/thumbs down technique, the secondary candidates’ approach to “exposing the discussion structure” distilled and restated the core axes upon which the discussion revolved. Doing so required candidates not only to track the lines of argument that students had raised, but also to organize these arguments in relation to the central questions of the lesson. Secondary candidates were more likely to expose the discussion structure than elementary candidates (*t*(30) = 2.16, *p* = .038). We discuss the implications of this finding below.

Only two elementary and two secondary candidates used meta commentary about orienting students towards each other that underscored the importance of listening and responding to their classmates. These comments took the form of subtle reinforcements, such as when candidate S2-8 said “I like that you referenced what Jamal said” before asking students to respond to the new point. Interestingly, “orienting to each other” was the only prong of the framework in which we did not observe differences between candidates who used instructor-created materials versus materials they created themselves.

### 5.4 Prong 4: Orienting to the discipline

Our findings indicate that both elementary and secondary candidates used over one quarter of their utterances to orient students to the discipline. The moves that fell under the prong of “orienting to the discipline” were those that distinguish disciplinary discussions in history from discussions in other subject areas, and included any utterance in which a candidate prompted or supported students to engage in the interpretative work of historical analysis.

Both elementary and secondary candidates oriented students to the discipline. Elementary candidates prompted students to source, contextualize, and corroborate the images in the lesson, and they also stabilized the content. Candidate utterances were coded as “sourcing” when they prompted students to consider a text's author or date, and coded as “contextualization” when candidates asked students to situate the text in its historical time and place. Half the elementary candidates used *both* sourcing and contextualization in their videos, and all but one of the remaining candidates either sourced or contextualized. Across videos, they enacted these moves in similar ways. In most cases, elementary candidates posed sourcing questions as closed elicitations (e.g., “When was this map made?“). On occasion, rather than ask students about the source, the candidate provided the information (e.g., “I did some research… Bartelomé de las Casas was a missionary who lived there at that time” (E1-4)). In rare instances, candidates asked interpretive questions about the source (e.g., “How do you know this artist was a colonist?” (E1-10)), but in two of the most interpretive instances (“Which of these images do you think is more accurate?” (E1-4) and “Which of these images do you think came first?” (E1-3)), the candidate posed the question as the lesson ended.

Elementary candidates used “contextualization” and “stabilizing the content” to orient students to deeper disciplinary understanding. In most cases, the instances of contextualization were linked to the lesson structure and initiated student engagement with the second, inferential stage of the visual inquiry lesson. The most effective of these invited students to imagine life at another time (e.g., “What do you think it was like to be a worker at this time?” (E1-1) or “What might have been important to the people who used this map in 1820?” (E1-5)). By contrast, teacher candidates stabilized content by providing information that could assist students in drawing accurate inferences, generally in response to student misunderstanding. For example, one candidate reminded students who were discussing maps of old auto assembly lines, “[Henry] Ford liked making the same kind of car every time—you didn't have any choice about the style or color of your car” (E1-1).

Among disciplinary moves, elementary teacher candidates most often prompted students to corroborate by asking them to compare two images. Elementary candidates had a far higher proportion of corroboration moves in their discussions than secondary candidates (*t*(30) = 3.22, *p* = .003), and those elementary candidates who used instructor-created materials were more likely to prompt students to corroborate (*t*(14) = 3.34, *p* = .005) than their classmates who used materials that they designed.

Secondary candidates oriented their students to the discipline of history at about the same rate as elementary teacher candidates. The three most commonly used disciplinary facilitation moves in the secondary videos were sourcing, contextualization, and stabilizing the content, in that order. Secondary candidates engaged their students in sourcing and contextualization approximately twice as often as elementary candidates, as measured by both the proportion of utterances and frequency per minute. Unlike in the elementary videos, however, corroboration moves were virtually non-existent.

We observed little consistency in how candidates used sourcing in the secondary videos. In most cases, instances coded as sourcing were relatively low-level questions that could be answered fairly easily. One candidate who facilitated a discussion about the Homestead Strike, for example, asked a series of closed questions that merely prompted students to identify the authors of the two documents and read the source notes out loud. In more rigorous examples of sourcing, one candidate prompted students to correctly attribute the quotes they pulled from the documents, and another posed a question about the audience of a document that required students to draw inferences about the author's purpose.

Only two candidates (S2-1 and S2-6) made sourcing the subject of the discussion. In other words, secondary teacher candidates rarely engaged students in open-ended, evaluative discussions about the reliability of the documents in the lesson. One of two teacher candidates who facilitated a discussion about source reliability relied heavily on instructional scaffolds to do this work. This teacher candidate focused their discussion on whether John Smith saved Pocahontas as depicted in the *Disney* movie, a lesson taken from the *Reading Like a Historian* curriculum (http://sheg.stanford.edu) that had been modeled for candidates early in the fall methods course. Over the course of the discussion, the candidate prompted students no fewer than four times to consider the reliability of the documents.

Secondary candidates used contextualization moves to ground students in the past as they interpreted the lesson's documents. For example, S2-7 responded to a student comment about how Cherokee viewed Indian Removal *before* the Trail of Tears by grounding students in the historic moment:

S: Either way, they're in a lose-lose situation… and he [Elias Boudinot, author of the Cherokee letter] would die too.

T: That's true but they don't know that yet.

S: They didn't know that yet, but if I were there, I would have chosen to leave, too.

Here, the candidate checked the student's impulse to use hindsight to evaluate the historical moment.

In other cases, candidates prompted students to contextualize using a fairly traditional recitation sequence. Three secondary candidates who contextualized most frequently used the move in conjunction with “marking text” to help students understand specific parts of the text. For example, S1-6 used a marking text move followed by contextualization as she helped students parse a historian's argument about the New Deal: “So I want us to turn to the text… look to the second paragraph. When he's talking about ‘excluding millions of Americans’ who specifically was excluded from receiving benefits? [student responds]… Yes, so African Americans and females. Why were they not receiving benefits?” In none of these cases did we observe candidates *returning* to these particular points later in the discussion or tying these understandings of the historical context to the central historical question.

Both elementary and secondary candidates were less likely to highlight and make explicit the discursive features of disciplinary discussion. The three disciplinary moves least observed were “highlight claim-evidence,” in which the candidate underscored the relationship between a claim and its evidentiary warrant, “modeling historical thinking,” in which the candidate demonstrated how to engage in disciplinary reasoning, and “disciplinary meta comment,” in which a teacher candidate articulated the nature, purpose, or importance of engaging in disciplinary discourse (see Table 2 for examples). Only two elementary utterances were coded as “disciplinary meta-comments.” E1-9 closed her lesson comparing two maps of Michigan with a clear disciplinary take-away: “Whenever you look at a map it's important to ask when is the map from and what does the person who made the map think is important.” Among the secondary candidates, only two made meta-comments on the purpose of disciplinary discussion. For example, when responding to a student who suggested the author did not have any proof to support his claim, she said, “[The author] doesn't have any *proof …* I like that! [addresses the class] So he's looking for *evidence*—good!”

### 5.5 Year 1 to year 2 comparison: the framework as instructional scaffold

In addition to serving as an analytical frame, the Framework for Facilitating Historical Discussions was an instructional material for secondary students in Year 2, which prompted us to look for differences between the Year 1 and Year 2 cohort. Although the cohorts were similar in most regards, we found that candidates in the second year were more likely than their predecessors from Year 1 to orient students to the discipline (*t*(14) = 2.61, *p* = .020) and to each other (37% versus 18% of utterances), though the latter finding was not statistically significant. Although they oriented to students to the text at a similar frequency, the Year 1 candidates were significantly more likely to do so by marking the text and asking students questions about particular passages (*t*(14) = 2.71, *p* = .017).

## 6 Discussion

This study contributes to an emerging body of literature seeking to capture the enactment of discussion facilitation by novices who have participated in practice-based instruction in their methods courses (Kavanagh & Rainey, 2017; Windschitl, Thompson, & Braaten, 2011). Though scholars have debated the value of using methods instruction to parse practice (Kennedy, 2016), little empirical research has assessed what candidates do when they attempt to enact these complex instructional practices with students. We examined how an instructional emphasis on facilitating discussion made its way into candidates’ field placements when they attempted to facilitate discussions with their students.

Our findings leave us optimistic. Despite the difficulty of facilitating discussions, our analysis suggests that novices can facilitate text-based discussions when they are prepared and supported. In both methods courses, we discussed, modeled, and rehearsed open-ended historical discourse. In their field placements, candidates posed open elicitations to engage students as sense-makers and initiate text-based historical discussions. Their students actively participated, in stark contrast with well-documented portraits of classroom discourse (e.g., Cazden, 2001; Nystrand et al., 2003; Saye & SSIRC, 2013). In those discussions, candidates across both programs oriented students to the text, to each other, and to the discipline as demonstrated by their instructors. The repetition of these moves shows novice teacher candidates animating the lesson structures they learned in their methods courses.

We further found that several of the scaffolds that we embedded in instruction may have assisted candidates in facilitating discussions. First, the structure of the assignment itself may have supported candidate enactment. Elementary candidates were instructed to use a prescribed question sequence in which they prompted students to notice and draw inferences about one image, to compare their observations to another image, and to use both images to answer a broader historical question. We found that, compared to secondary candidates, they were more likely to prompt their students to notice, draw inferences, and corroborate the images in the lesson. These findings offer a powerful argument for providing novices with a concrete discursive structure to organize and sequence their discussion facilitation (see Ghousseini, Beasley, & Lord, 2015 for a comparable example in math).

Secondary candidates were not provided an analogous question sequence, which may account for differences in disciplinary moves that we observed between programs. For example, only two secondary candidates prompted students to corroborate texts, even though almost all their lessons included multiple documents, while nearly all the elementary candidates prompted students to corroborate. Similarly, many of the elementary candidates' prompts to contextualize were aligned with the second stage of the visual inquiry lesson, whereas secondary candidates' efforts were more haphazard and rarely invited students to imagine broadly what life might have been like at the time under study. These findings suggest that the question sequence and structure provided in the visual inquiry lesson may have scaffolded both students' interpretation of the images and teacher candidates’ efforts to engage students in disciplinary discussion.

At the same time, the particular sequence of the visual inquiry lesson—moving from observation to inference to interpretation—may have de-emphasized the central question and obscured the learning goal. Only two of the 16 elementary candidates attempted to engage students in historical interpretations of the images with sufficient time remaining in the lesson. Secondary candidates were more likely to pose the central historical question and prompt students to interpret the documents, likely because the document-based lesson structure foregrounded a central historical question. That secondary candidates were significantly more likely to expose the discussion structure than elementary candidates may also relate to the lesson structure: as students weighed in on the central question, secondary candidates may have been inclined to help students track the key arguments being raised. Still, secondary candidates would likely have benefitted from more scaffolding, given that nearly half did not pose the central historical question.

We also found that the *materials* upon which candidates base discussions matter. Secondary candidates facilitated their discussions around written texts rather than visual images, and they were more likely than elementary candidates to prompt students to summarize, source, and contextualize the documents. Additionally, their prompts to contextualize were often tied to specific passages of the texts. These findings suggest that candidates worked to ensure document comprehension and interpretation in ways that elementary candidates, using visual images, did not. We further found that the use of instructor-created materials supported candidate enactment, specifically around orienting students to the text. Elementary candidates who used instructor-created materials were more likely to mark the text and corroborate; secondary candidates who used instructor-created materials were more likely to prompt students to support their claims with textual evidence.

We can suggest three reasons instructor-created materials might support candidates' engagement with text. First, instructors might select higher quality texts that reward repeated investigation. Second, it is possible that when candidates are provided with classroom-ready materials, they are able to devote more attention to implementation and enactment. Instructor-created materials could lessen the cognitive load involved in planning and help candidates be more prepared to facilitate discussions. Third, it could be that candidates are more willing to turn students’ attention to texts in instructor-created materials because they are more confident that students will find generative evidence for their claims.

Finally, we found significant differences between secondary candidates in Year 1 and Year 2, when the teacher educator explicitly incorporated the Framework for Facilitating Historical Discussions into methods instruction. The higher proportion of marking text moves in Year 1 is consistent with our previous assessment (Reisman et al., 2018) that Year 1 candidates focused more on orienting students to text than on the other prongs of the framework. That Year 2 candidates were significantly more likely to orient students to the discipline is consistent with the instructional emphasis in the second year of methods instruction. This finding suggests, as others have argued (Grossman et al., 1999), that conceptual tools may help mediate candidates’ development, in this case broadening their understanding of the purposes of discussion and the range of facilitation moves available to them.

In one sense, this analysis paints a hopeful picture. Teacher candidates in our study often made moves characteristic of disciplinary discussions (e.g., Kazemi & Cartun, 2016), particularly when leaning upon scaffolds provided for them by practice-based methods courses. Additionally, the specifications of practice aided our observation of both elementary and secondary discussions, suggesting the existence of stable and observable instructional practice across age groups. Yet seen from a wider lens, it is not clear that teacher candidates provided meaningful disciplinary learning experiences for students. In both contexts, discussion was intended to help students understand the interpretive nature of historical analysis and engage with the content in response to a central question. The majority of teacher candidates did not provide students the opportunity to build these understandings. Though they posed open elicitations, many struggled to facilitate student discourse around the central interpretive questions of the lesson. Many secondary candidates focused their text-based prompts on summarization rather than on interpretation or argumentation. Even though both elementary and secondary candidates made efforts to orient students to each other, they struggled to engage students in collective knowledge construction. Finally, across all prongs, candidates in both programs did not provide meta-commentary on the importance or purpose of engaging in various practices.

The general absence of meta-comments suggests that candidates struggled to comment *upon* the discussion as it was unfolding. Meta-comments require candidates to decide to pause the conversation, comment on how to be a better discussion participant or historian, and then restart the conversation. This sequence demands exceptional clarity about the goals of the discussion and the range of potential student comments. Several other infrequently used moves, such as highlighting claim-evidence relationships and modeling disciplinary thought, also depend on the candidates’ ability to identify a teachable moment and interrupt the conversation without prior scripting. These moves remain targets for the future development of candidates.

We consider this composite portrait of novice discussion facilitation a representation of emergent practice. The teacher candidates in these videos vary in their style and skill as facilitators, but as a group, they are beginners—awkward dancers, staring at their feet as they learn the moves. In a teaching landscape where even established teachers sometimes find it daunting to facilitate open-ended and disciplinarily rich discourse, we are buoyed by these earnest attempts.

### 6.1 Implications

Our optimism over these findings is tempered by our acknowledgement of the study's limitations. As anyone involved in teacher evaluation would caution, a single observation can hardly offer a reliable assessment of instruction—even more so for our sample, where the videos constituted neither a full lesson nor a random sampling. Our analysis of the videos would have been more robust had we coded student comments, many of which were unfortunately inaudible or indecipherable. Ultimately, the strength of this research program will be connecting practice-based teacher education to evidence of student learning. Future research should also consider longitudinal designs that capture candidates' practice in their first years of teaching and beyond.

Our analysis also raised questions about analyzing discussions at the level of the utterance. The nature of our video data precluded our coding discussion facilitation at a broader grain size (e.g., framing or closing discussion). Few of our videos reflected intact activity structures with clear-cut beginnings, middles, and ends, and even if they had, the structures would have varied not only between elementary and secondary candidates but also among the secondary candidates. Still, we are exploring how we might better assess whether teacher candidates provided opportunities for their students to develop deeper understandings over the duration of a discussion. Future efforts to assess the nature of teacher candidates’ historical discussions must use both narrower and wider interpretive lenses.

The fact that many teacher candidates in our study enacted moves across the four prongs of the framework and yet struggled to engage students in meaningful disciplinary discussion suggests that teacher candidates might have benefitted from a clearer articulation of the overall purpose of disciplinary historical discussion. The contrast with science (see Kloser et al., in press) is striking. By naming the analogous core practice in science “*sensemaking* discussions,” these science education researchers assign an undeniable purpose and learning goal for collective discourse, namely, to explain complex scientific phenomena (e.g., Why do astronauts experience weightlessness?). We might consider what would constitute an analogous purpose for discussion in history. This purpose might then serve as a further instructional scaffold—a more conceptual one—that would help teacher candidates stabilize their footing as enactors of disciplinary historical discussion.

One way to scaffold candidates’ emergent attempts to lead purposeful disciplinary discussions might be to identify the range of questions that are posed in historical discussions and the kinds of reasoning that each invites. For example, teachers might pose causal questions (e.g., Why did X happen?), investigative questions (e.g., Did X happen?), evaluative questions (e.g., Was X justified, given what people knew at the time?), descriptive questions (e.g., How did X change over time?), or even, at a smaller grain size, sourcing questions, (e.g., Do you find X to be a reliable source?). Though hardly comprehensive, one can imagine that offering candidates a taxonomy of historical questions might help them specify their learning goal, design clearer prompts, and listen for and highlight the most relevant or generative student comments. For example, E2-3 asked the following question about the Carlisle Boarding School for Native Americans: “How do these pictures show how entering the school changed or affected their culture?” This question was primarily descriptive—she wanted students to identify differences in before/after photographs of Native American children who attended the boarding school. However, her ultimate goal was for students to infer causes for the change, a goal that could have been achieved with a more straightforward causal question (e.g., Why did their appearances change?). The discussion would have been an opportunity for students to infer what happened in the schools and why such schools might have been established in the first place, at which point the candidate might have posed an evaluative question (e.g., Were such schools cruel, given what people thought and knew at the time?). Each type of question would presumably invite different forms of reasoning about evidence and about the past.

Just as teacher candidates' emergent practice showed both promise and room for growth, our efforts represent a first attempt at capturing how teacher candidates enacted historical discussions in the context of practice-based methods instruction. We found that our Framework for Facilitating Historical Discussions aided our understanding of how teacher candidates facilitated historical discussions. Furthermore, it may have supported teacher candidates' enactment as an instructional scaffold, as evidenced by secondary Year 2 teacher candidates' frequent attempts to orient students to each other's ideas. At the same time, we believe that the Framework would be a more useful tool if the learning goals of disciplinary historical discussions were more carefully specified. We wonder if doing so might refine our analysis of teacher candidates' emergent attempts to facilitate historical discussion. We invite our colleagues to join us in further conceptualizing the work of discussion facilitation so that we might support novice teachers' engagement in this valuable instructional work.

## Funding

This study has been conducted as part of the work of the Core Practices Consortium (corepracticeconsortium.com) and builds on the contributions of its members. This collective work has been supported by the Bill & Melinda Gates Foundation under Grant OPP1089179 and the Spencer Foundation under Grant 201600110.
